# A Phenomenological Approach to Financial Toxicity: The-Economic-Side Effect of Cancer

**DOI:** 10.3390/curroncol31100454

**Published:** 2024-10-11

**Authors:** Nicolò Panattoni, Emanuele Di Simone, Erika Renzi, Flavia Di Carlo, Fabio Fabbian, Marco Di Muzio, Annalisa Rosso, Fabrizio Petrone, Azzurra Massimi

**Affiliations:** 1 Department of Public Health and Infectious Diseases, Sapienza University of Rome, 00185 Rome, Italy; nicolo.panattoni@uniroma1.it (N.P.); emanuele.disimone@uniroma1.it (E.D.S.); azzurra.massimi@uniroma1.it (A.M.); 2 Nursing, Technical, Rehabilitation, Assistance and Research Direction, IRCCS Istituti Fisioterapici Ospitalieri (IFO), 00144 Rome, Italy; dicarlo.1848509@studenti.uniroma1.it (F.D.C.); fabrizio.petrone@ifo.it (F.P.); 3 Department of Medical Sciences, University of Ferrara, 44121 Ferrara, Italy; fbbfba@unife.it; 4 Department of Clinical and Molecular Medicine, Sapienza University of Rome, 00189 Rome, Italy; marco.dimuzio@uniroma1.it; 5 Department of Environmental and Prevention Sciences, University of Ferrara, 44121 Ferrara, Italy; annalisa.rosso@unife.it

**Keywords:** financial toxicity, cancer patients, caregivers, phenomenological study

## Abstract

The economic burden of chronic diseases such as cancer could negatively impact patients’ health and quality of life. The daily management of the disease results in economic needs that patients often face directly, which may lead to real toxicity, just defined as financial toxicity. This study aims to explore cancer patients’ experiences, emotions, opinions, and feelings related to the phenomenon of financial toxicity. A phenomenological qualitative descriptive study was conducted through face-to-face interviews with adult oncological patients. The sample (*n* = 20) was predominantly composed of females (with a meanly 58 years old) with breast cancer and in chemotherapy treatment. The most relevant topics that emerged from the patients’ experiences were the impact on work, the distance from the treatment centre, the economic efforts, the impact on the quality of life, and the healthcare workers’ support during the healthcare pathway. From the phenomenological analysis of the interviews, three main themes and seven related subthemes emerged. This study provided a phenomenological interpretation of financial toxicity in adult cancer patients and underlines that this issue involves families or caregivers, too. Financial problems appear relevant for those who experience cancer and should be included in a routine assessment by healthcare professionals.

## 1. Introduction

The term financial toxicity (FT) refers to a complex and multidimensional phenomenon relating to a disease’s economic burden and its negative impact on health outcomes and quality of life (QoL) [1]. In the context of oncological diseases, FT indicates the economic burden resulting from cancer treatments (e.g., chemotherapy, surgery) [2], leading to treatment failure [3], decreased adherence to the therapeutic plan, worsening of QoL [4,5], and sometimes to forego cancer care [6]. Direct costs (the cost related to medical expenses), indirect costs (such as lost wages) and psychosocial aspects (for example, anxiety, stress and depressive symptoms) due to the financial situation [4] all contribute to FT in cancer patients. Available evidence suggests that compared to other disease types, the economic burden in cancer is higher [7,8,9] due to more significant needs in terms of screening investigations and accurate diagnosis, multidisciplinary care and longitudinal follow-up [10].

Cancer-related FT is a global problem, reported also in countries with a universalistic National Health Service (NHS), suggesting that the phenomenon is not only linked to the direct costs of anti-tumour treatments but also to several secondary factors that significantly impact patients and their families’ lives [11]. For these reasons, it is necessary to identify preventive strategies for patients and healthcare personnel to minimise FT, promote access to equitable treatment, and reduce health disparities [12]. As the literature suggests, most patients would be interested in discussing the financial aspect of their illness, even though this topic is often not addressed unless the patient declares the issue expressly [13], suggesting the need for a patient’s experience exploratory approach to address it. A phenomenological qualitative methodology seems the best way to acquire the patients’ internal knowledge of experiences, perceptions, emotions, judgements, perspectives, and visions of FT. Therefore, we conducted a phenomenological descriptive study to explore the phenomenon of FT through cancer patients’ experiences, emotions, opinions, and feelings to gather helpful information to design potential doable preventive strategies or interventions [14].

## 2. Materials and Methods

### 2.1. Design

According to Husserl’s philosophical perspective [15], a phenomenological qualitative descriptive study [16] was performed. This phenomenological design was chosen to acquire the patients’ internal knowledge of the FT phenomenon’s experiences, perceptions, emotions, judgements, perspectives, and visions as they perceive and interpret it, without researchers’ external judgements or interpretations [15]. This method seems to be one of the best ways to simplify and enable the complexities of medical conditions, emotions, and life experiences through a free and nonjudgmental approach, performing open-ended interviews to bring about patients’ subjective and intuitive information through the tales of experiences and feelings and give appropriate meaning to their words [17]. The results of this type of study help the researchers understand one another’s world with the feelings of those who describe it and use these findings to personalise possible future interventions [15,16]. The COREQ Guidelines (Consolidated Criteria for Reporting Qualitative Research) were used to improve the study’s consistency, quality, and rigour (Table S1) [18].

### 2.2. Participants

After agreeing to participate and signing the informed consent, a consecutive sample of cancer patients was interviewed until data saturation [19]. After each interview day, a saturation grid was used to define the enrollment deadline based on the data saturation criteria, that is, the absence of significant new information provided by new interviewees [19]. Indeed, data saturation occurs when additional data do not emerge in any new theme [20] or when all new information collected is redundant in the already collected data [21]. According to Sandelowski’s sampling strategy to promote equality and quality in data collection [22], patients were recruited consecutively according to the following inclusion criteria: adult patients (age > 18 years), patients diagnosed with cancer, patients without difficulties in verbal communication, and signed consent to participate in the study. All patients without a confirmed cancer diagnosis and patients with cognitive impairment, psychiatric disorders, or poor compliance with study procedures were excluded. All participants received necessary explanations about the study they were participating in and the interview methods.

### 2.3. Setting and Data Collection

Data were collected through open-ended interviews between June and August 2023 at the two oncology Day Hospital units of an Italian oncological research institute by nurse researchers. Face-to-face interviews were conducted during the patients’ daily hospitalisation in a separate room free from external noise and distractions. The interviews lasted 3 to 10 min and were digitally audio-recorded and transcribed verbatim. Before starting the interview, the main objectives and topics of the study were discussed, and the interviewer encouraged the participant to have maximum freedom in answering the questions. Subsequently, informed consent was signed. The interview included eight questions constructed ad hoc to discuss the study’s most important topics, leaving the patient free to direct the communication flow (Table 1). No pre-test was performed on the interview guide by researchers to ensure spontaneity and freedom in the answers during the interviews.

### 2.4. Data Analysis

According to Giorgi’s descriptive phenomenological method [23] and an inductive content analysis process [24], a text analysis was performed on the participants’ stories collected. This method consists of five basic steps [23]: collection of verbal data, data reading, data decomposition into units, data organisation and expression in a disciplinary perspective, and data synthesis for the aim of expressing the phenomenon essence.

Therefore, initially, a researcher recorded the interviews (F.D.C., RN), and a second researcher (E.R., RN, MSN, PhD) transcribed them verbatim, including pauses, gestures and emotions (highlighted in the field during the interview) so that the meaning of the interviewees’ words was preserved. The second phase involved reading the transcripts with an approach free from preconceptions to obtain a general sense of the information collected without attempting to thematise it. The third step of analysis involved dividing the data collected into “units of meaning”, i.e., distinct units that express an autonomous meaning [23], after rereading the transcript word by word. In the following phase, each unit of meaning was analysed and reorganised from a disciplinary point of view with a clinical point of view. Finally, the last phase allowed the data to be synthesised by grouping the units of meaning into categories to summarise the meaning of the phenomenon’s essence. In the final moment of abstraction, subcategories with conceptual and semantic similarities were grouped into macro-categories. Thus, the “units of meaning” were understood as essences, and their relationships were made explicit through themes and subthemes. A third researcher (N.P., RN, MSN, PhD) experienced in qualitative research oversaw all analysis phases and discussed all contrasts until a unanimous agreement was reached. In this study, the researchers did not use specific qualitative data analysis software; they used grids and tables constructed for each phase’s analysis and group discussion.

### 2.5. Trustworthiness

The Lincoln and Guba criteria [25] were adopted to guarantee the results’ trustworthiness, credibility, reliability, confirmability, and transferability. To ensure credibility, the sampling enrollment was continued until data saturation. Reliability was guaranteed with the triangulation technique, which involves the participation of two or more researchers during the analysis to agree with the different perspectives of the results, adding largeness to the examined phenomenon and multiple conclusions [25]. Audit trails guaranteed confirmability, where two researchers who did not participate in data collection and analysis re-examined the survey process to obtain equal results. Then, the transferability was guaranteed via an accurate and precise description of the methodology, the research process, and the results.

### 2.6. Ethical Considerations

The study was approved by the Central Ethics Committee of IRCCS Lazio (Experimental Registry No. 1873/23 of 30 May 2023) and was conducted according to the Declaration of Helsinki [26]. Participants were asked to sign a written informed consent, and the anonymity of the information collected was guaranteed. The data transcription and analysis process ensured that no participant could be traced by the researchers after the data collection. They were also informed that their contribution to the study was voluntary and without compensation.

## 3. Results

### 3.1. Participant Characteristics

A consecutive sample of 20 cancer patients was enrolled in the study. None of the participants enrolled in the sample were lost during the data collection phases, and all 20 patients completed the interview. The sample consisted predominantly of females (*n* = 16; 80%), with an average age of 58 years (range 34–76). The most represented neoplasm was breast cancer (*n* = 8; 40%), followed by lung cancer and liver cancer (both *n* = 3; 15%). All participants were undergoing chemotherapy treatment (*n* = 20; 100%). Table 2 describes the demographic characteristics collected.

### 3.2. Phenomenological Findings

**Figure 1:**
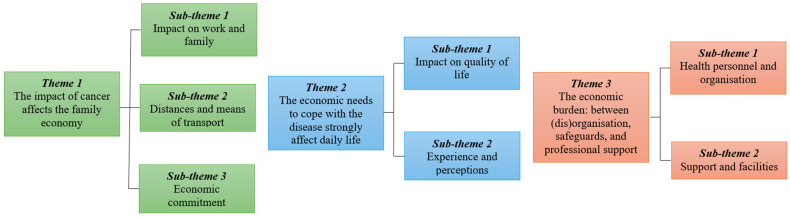
Thematic map illustrating the phenomenological themes and subthemes.

The interviews’ phenomenological analysis [19,20] revealed three main themes and seven related subthemes, as shown in Figure 1.

The emerging themes allowed us to understand the FT experience and the perspective of patients’ families and caregivers.

Table 3 shows each participant’s detailed prevalence of the major themes (as suggested by Newberry [27]), highlighting that cancer impacts the family economy for 14 out of 20 participants. Although our study’s analysis and objective were not focused on identifying differences in impact based on different cancer types, the information in Table 3 provides a prevalence breakdown of significant cancer-related themes.

We provide a presentation of the themes and subthemes supported by the verbatim extracted from the interviews: the patient reporting the sentence was coded by a patient code (P1–P20), sex (M = male, F = female), and age, e.g., (P11, F, 48 years old).

#### 3.2.1. The Impact of Cancer Affects the Family Economy

It is now known that the economic burden associated with the oncological care pathway could cause severe difficulties for the patient and his family. Indeed, a cancer diagnosis can have significant consequences both for the psycho-physical viewpoint and for the patient and family’s economy. As emerged from interviews, this hardship includes the direct costs of the disease (such as the costs of medicines or private medical visits) and all indirect expenses, such as travel to the treatment centre or money lost due to reduced working hours. These oncological diseases’ “side effects” can affect the family economy several years after diagnosis. This theme can be explained with three subcategories: (1) impact on work and family, (2) distance and means of transportation, and (3) economic commitment.

Many patients reported having lost or reduced their working hours, temporarily or permanently, after their cancer diagnosis. This is both due to the physical limitations faced by patients undergoing chemotherapy, such as asthenia and nausea, which challenge the capacity to sustain a working day and to the time that an oncological treatment process takes away from patients each week.

*“[...] Unfortunately, I work in catering, a very physically demanding job. Although I realise that an entrepreneur cannot take on the burden of someone absent from work for more than six months, it would have been difficult for me to return to work during chemotherapy. There are days when you just can’t physically do it. [...].”*

(P1, F, 47 years old, breast cancer).

*“I hope I don’t lose my job...of course, I can work less these days [...].”*

(P8, F, 57 years old, breast cancer).

Other patients reported that their family members also had to miss working days to sustain them on their treatment journey. This issue, in addition to requiring additional efforts and organisational commitment of the patients’ families, often results in lower takings.

*“(…) [...] my wife. Reduce working hours...today, for example, she accompanied me and did not go to work. [...].”*

(P3, M, 62 years old, lung cancer).

Distance from the treatment centre is a part of FT. Some patients are treated in cities or regions that differ from their own. This affects their QoL and economic situation due to complex family planning and the time patients and their families or caregivers must spend in a different city. Indeed, patients reported numerous expenses related to transportation costs, such as car maintenance and management, highway tolls, taxis, train tickets, and accommodation during the treatment pathway. Therefore, the treatment centre’s proximity to the people could improve their quality of life, promote economic savings, and mitigate FT.

*“It takes me about an hour [to get to the treatment centre]... and of course, I have my car so, the cost of petrol. They don’t give it to you for free because you must do chemotherapy, I mean.”*

(P10, F, 63 years old, liver cancer)

*“[...] I reach the health centre *via* the motorway because I come from another city [...] so the cost of petrol and the motorway...”*

(P11, F, 34 years old, breast cancer).

Patients have to bear many expenses related to oncological disease. These include the costs of private medical visits and exams that many are forced to do to respect the times foreseen by the treatment path (given the long waiting lists in the NHS), medicines not provided by the NHS, and all expenses that imply a better QoL, such as additional supplements or the purchase of a wig.

*“Yes, many expenses: for private CT scans, for analyses, for things I had to do urgently, and I didn’t have availability through the National Health Service promptly. I had to do them privately, as well as the MRI.”*

(P18, F, 67 years old, lung cancer).

Indeed, some of them report having had to ask their family members for financial help to cope with this financial burden.

*“[...] I am just above [financially] being able to treat myself properly because if I didn’t have the support from my family... I wouldn’t have been able to afford the car to get to the treatment centre, nor the wig to maintain a ‘non-sick’ look and many small supplements that still help you feel better.”*

(P1, F, 47 years old, breast cancer).

#### 3.2.2. The Economic Need to Cope with the Disease Strongly Affects Daily Life

This theme is divided into two subcategories (impact on QoL and experience and perceptions). It addresses essential topics highlighting how FT has systemic consequences, impacting all spheres of patients’ lives. It places a high importance on their QoL and their perceptions of the disease they face. 

From the interviews, FT is considered a significant side effect of oncological treatment that can significantly reduce the QoL of patients and their families. Many patients told us that due to the expenses caused by the disease, they have been forced to change their standard of living, giving up recreational activities, education, culture, or free time.

*“[...] I have changed, or rather at this time, I am changing my standard of living, according to the [financial] expenses related to my illness”*

(P3, M, 62 years old, lung cancer).

Patients, even those with no financial difficulties, clearly became aware of their illness and the economic means necessary to deal with it. Some patients expressed their worries about the future due to possible economic problems arising from the disease.

*“(…) for me there are no problems but for other people they are very big problems. (…) this is an important disease”*

(P10, F, 63 years old, liver cancer).

#### 3.2.3. The Economic Burden: Between (dis)Organisation, Safeguards, and Professional Support

Another relevant topic that emerged from patients’ experiences is the role of healthcare professionals in managing the economic burden.

In fact, according to patients’ stories, a good healthcare organisation linked to adequate emotional support is essential for the success of a therapeutic process. In terms of financial needs, knowing how to advise and protect the patient is crucial in mitigating the costs related to the disease.

Most of those interviewed told us that they were satisfied with their treatment path, especially with nursing management, from a strictly professional and relational point of view.

*“Nurses are very good professionals...nurses are amazing, in my opinion.”*

(P10, F, 63 years old, liver cancer).

Instead, some patients have complained of a lack of communication with physicians, reporting difficulties establishing an empathic relationship. This situation increases the burden on people already affected by the disease.

*“Doctors don’t realise that they’re talking to people who are scared and don’t realise what they’re saying”*

(P1, F, 47 years old, breast cancer).

Finally, the problem of long waiting lists in our NHS emerged, with many patients turning to private services (i.e., *intramoenia*), which consequently increased out-of-pocket spending.

*“I am satisfied with the path I am taking with you sanitary people. That’s all I can say...sure, the waits are long in some situations [...].”*

(P8, F, 57 years old, breast cancer).

From what emerged from the interviews, healthcare staff, especially nursing staff, proved to be willing to help patients, not only from an emotional point of view but also in managing appointments for visits and examinations, trying to facilitate them where possible, given the long waits for the NHS.

*“They tried to make me do everything in the facility and not privately”*

(P1, F, 47 years old, breast cancer).

*“All the possible contribution from a professional point of view”*

(P3, M, 62 years old, lung cancer).

## 4. Discussion

The themes and subthemes of the phenomenological analysis allowed us to achieve the study objective, which aimed to explore the phenomenon of FT through cancer patients’ experiences, emotions, opinions, and feelings. International interest in evaluating the association between FT and QoL is growing, considering the patient and his family unit [28,29,30]. To our knowledge, only another study published in the international literature [26] explored FT in cancer patients with a phenomenological methodology; however, it did not explore the phenomenon in the patient’s family or caregivers. In our study, most of the patients interviewed reported discomfort and difficulties in dealing with the expenses resulting from their cancer diagnosis. Furthermore, it emerged that FT has a negative impact not only on the individual patient’s QoL but also on the entire family unit. We also analysed how healthcare personnel may support or facilitate patients concerning FT during their clinical pathway.

“The impact of cancer on the family economy” (the first main theme that emerged) impacts the entire family economy, affecting the QoL not only of the patient but also of his/her family/caregivers. As already enlightened in several studies [31], after their cancer diagnosis, many patients noted that they had to reduce their working hours or those of their family members, which often resulted in fewer takings. Furthermore, some patients reported difficulties in regularly reaching the place of treatment due to the distance from their homes. Increasing new technologies could mitigate out-of-pocket costs due to the distance from home to the care centre [32,33], a relevant matter also reported by our sample. Indeed, most of those interviewed said they were uncomfortable with the costs of travelling to the treatment centre due to car maintenance, the cost of petrol or other means of transport.

In general, the patients interviewed reported fear of not being able to manage all the direct and indirect expenses related to the oncological disease. These results, relating to a particular dimension of FT, are in line with the literature: a recent study from Minnesota suggested that the out-of-pocket expenses for adhering to oncological treatment and daily travel to the treatment centre have an essential effect on FT with a negative impact in terms of overall survival and QoL [34].

As Abrams and colleagues have already reported [12], the need for patients to change their lifestyle is relevant because it occurs when coping with the disease’s costs. The perspective related to “The economic need to cope with the disease strongly affects daily life” was reported by patients: they had to give up some activities that inevitably contributed to achieving a good QoL (such as restaurant dinners, gym memberships, or holidays) since they were now incompatible with their economic priorities or possibilities.

Furthermore, through the interviews, the patients realise the importance and economic weight that lies behind their pathology and show fear towards the future.

The third and last theme was “The economic burden: between (dis)organisation, protection and support of professionals”. The organisation of the NHS and the support of healthcare professionals play a fundamental role in managing costs related to the disease and patient satisfaction with the oncological therapeutic pathway. Many patients interviewed felt satisfied with their treatment path, praising the professionalism and availability of healthcare personnel, especially nurses, even though some critical issues emerged regarding the therapeutic relationship and communication between physicians and patients. A recent Australian study suggested that general practitioners could play an essential role in the team to foster better communication, relationships, collaboration between specialist teams, and greater health literacy on cost issues [35]. Furthermore, the lack of communication between doctors and patients, which often translates into the inability to establish an empathic and trusting relationship, seems to increase the phenomenon of FT [4].

In Italy, the understanding of the cancer-related FT phenomenon is rising due to the interest in studying the dynamics of economic burden in a country where the NHS is publically funded [36,37]. In this regard, a study by Riva and colleagues [11] developed a specific tool to assess cancer-related FT in a country with a fully public NHS. The establishment of a “financial navigator”, already present in some other countries [38], to guide and support the patient in managing the financial aspects related to the clinical pathway could mitigate the phenomenon of FT, decreasing the burden resulting from cancer diagnosis [38]. Finally, considering an individual’s economic status is one of the Social Determinants of Health (SDoH), healthcare workers involved in patient care pathways should consider FT a relevant implication in the oncology population to improve patient outcomes and QoL [39]. Our thematic analysis highlights how specific policies are still needed today to offset the weight of the direct and non-direct costs of diagnosis and oncological treatment in this vulnerable population.

### Limitations

This study has some limitations. First, the main limitation is linked to the generalizability of the data because the phenomenological approach adopted, although reliable [25], is related to the subjective experience of the enrolled patients. We therefore recommend caution when generalising our findings to other oncological populations. Furthermore, our patients were undergoing chemotherapy, so we do not recommend generalising our findings to a population of cancer-survivor patients. In addition, our sample was mostly females with breast cancer, and the age groups varied from 34 years old to 63 years old, so we recommend considering that the patients’ experiences regarding finance could be different. Conducting further studies on this population will provide interesting information on the effects of a long-term FT experience, such as roles and responsibilities in the clinical management of FT [40] or financial literacy education programmes [41].

## 5. Conclusions

To our knowledge, this is the first published study providing a phenomenological interpretation of FT that also reveals the impact on the patients’ families or caregivers.

Our inductive descriptive analysis of the data produced three exciting themes: “The impact of cancer affects the family economy”; “The economic need to cope with the disease strongly affects daily life”; and “The economic burden: between (dis)organisation, safeguards, and professional support”. The topics most discussed in the interviews were the impact on work, the distance from the treatment centre, the economic commitment of the oncological disease, the impact on the QoL, and the support and benefits provided by healthcare personnel during the treatment process.

Future research based on mixed-method studies (for example, through meta-inference) could help to compare objective FT assessment data with subjective experience directly reported by the patient. Furthermore, contextualisation with cancer registry data and trends in cancer diagnosis incidence could improve qualitative findings. It could be helpful to support healthcare decision-makers who seek to identify patients at increased risk of FT already at diagnosis to immediately implement all necessary interventions to mitigate the problem and reduce the financial burden of the disease.

## Figures and Tables

**Table 1:** Interview guide.

Main Topics	Questions
FT experience on oneself	1. Does your economic situation allow you to take care of yourself?
	2. Is your home far from the treatment centre? Do you have to spend money to get there? (means of transport/accommodation)
	3. Are you afraid of losing your job or not being able to work because of your illness?
	4. Have you incurred expenses for medications, supplements, and private medical visits?
	5. Have you had a reduced quality of life to deal with the expenses of the illness (e.g., food, holidays, restaurants)?
FT experience related to family or caregivers	6. Has your illness affected your finances or your family’s finances?
	7. Has a family member had to frequently take time off work or reduce their working hours to accompany you in your treatment journey?
FT experience related to healthcare workers	8. Did the healthcare staff facilitate your treatment pathway?

**Table 2:** Participants’ demographic characteristics (N = 20).

Patient Characteristics	N
**Age, mean ± SD (range)**	58 ± 10.95 (34–76)
<65 years	14 (70%)
≥65 years	6 (30%)
**Gender**	
Male	4 (20%)
Female	16 (80%)
**Cancer type**	
Breast cancer	8 (40%)
Lung cancer	3 (15%)
Liver cancer	3 (15%)
Prostate cancer	1 (5%)
Peritoneal carcinomatosis	1 (5%)
Stomach cancer	2 (10%)
Colorectal cancer	1 (5%)
Bladder cancer	1 (5%)
**Chemotherapy**	
Yes	20 (100%)
No	0 (0%)

**Table 3:** Prevalence of themes: number of cancer-related quotes per theme and participants.

	The Impact of Cancer Affects the Family Economy	The Economic Need to Cope with the Disease Strongly Affects Daily Life	The Economic Burden: Between (dis)Organisation, Safeguards, and Professional Support
Breast cancer	P1, P4, P11, P14, P15, P16	P1, P14	P1, P14, P15, P16
Lung cancer	P3, P18	P3	P3, P12, P18
Liver cancer	P19	P19	P10
Prostate cancer	-	P2	-
Peritoneal carcinomatosis	P20	P20	P20
Stomach cancer	P7, P17	-	P7, P17
Colorectal cancer	P13	-	P13
Bladder cancer	P6	-	P6

Note: P1–P20 = patient code.

## Data Availability

The original contributions presented in the study are included in the article/Supplementary Material. Consolidated Criteria for Reporting Qualitative Research (COREQ) guidelines were used for the reporting [15].

## Supplementary Materials

The following supporting information can be downloaded at: https://www.mdpi.com/article/10.3390/curroncol31100454/s1, Table S1: COREQ (COnsolidated criteria for REporting Qualitative research) Checklist.
